# Research Progress of M13 Bacteriophage-Based Biosensors

**DOI:** 10.3390/nano9101448

**Published:** 2019-10-11

**Authors:** Jong-Sik Moon, Eun Jung Choi, Na-Na Jeong, Jong-Ryeul Sohn, Dong-Wook Han, Jin-Woo Oh

**Affiliations:** 1 National Core Research Center for Hybrid Materials Solution, Pusan National University, Busan 46241, Korea; 2 Department of BIT Fusion Technology Center, Pusan National University, Busan 46241, Korea; eunjung721203@gmail.com; 3 Department of Public Health Sciences, Graduate School, Korea University, Seoul 02708, Korea; nana8931@naver.com (N.-N.J.); sohn1956@korea.ac.kr (J.-R.S.); 4 Department of Cogno-Mechatronics Engineering, College of Nanoscience & Nanotechnology, Pusan National University, Busan 46241, Korea; nanohan@pusan.ac.kr; 5 Department of Nanofusion Technology, College of Nanoscience & Nanotechnology, Pusan National University, Busan 46241, Korea

**Keywords:** virus, biosensor, M13 bacteriophage, color sensor, phage display

## Abstract

Recently, new virus-based sensor systems that operate on M13 bacteriophage infrastructure have attracted considerable attention. These systems can detect a range of chemicals with excellent sensitivity and selectivity. Filaments consistent with M13 bacteriophages can be ordered by highly established forms of self-assembly. This allows M13 bacteriophages to build a homogeneous distribution and infiltrate the network structure of nanostructures under mild conditions. Phage display, involving the genetic engineering of M13 bacteriophages, is another strong feature of the M13 bacteriophage as a functional building block. The numerous genetic modification possibilities of M13 bacteriophages are clearly the key features, and far more applications are envisaged. This paper reviews the recent progress in the application of the M13 bacteriophage self-assembly structures through to sensor systems and discusses future M13 bacteriophage technology.

## 1. Introduction

### 1.1. M13 Bacteriophage

Distinguishing infinitesimal amounts of chemicals or microbials in a precise and simple manner is one of the most important techniques in academia and industry. Classical sensor technology is categorized as electromagnetic (gamma radiation, optics, microwave, radio wave, and Eddy current), mechanical (sound, MEMS, and fluid), magnetic, chemical (affinity, catalytic reactions, electrochemistry, and biochemistry), nuclear (nuclear magnetic resonance), and a combination of them (opto-acoustics and membrane technology) [1,2]. Generally, the key features of sensor technology are the low cost, small size, robustness, selectivity, and sensitivity [1,3].

Recently, the use of ecofriendly materials has been one of the main points in research and industrial manufacturing. The M13 bacteriophage, which can be categorized as a natural polymer, has been a major research focus owing to its target-specific response in chemical reactions with outstanding sensitivity [4]. The bacteriophage is a human-safe viral material that only infects certain criteria of bacteria, such as *Escherichia coli.* [5].

**Figure 1:**
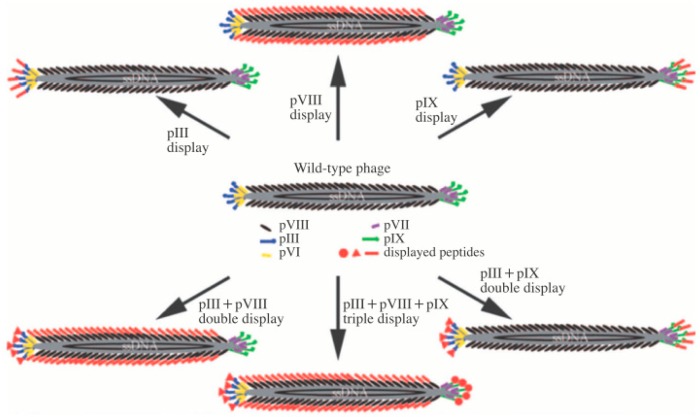
Basic structure of the M13 bacteriophage and the possible pathway of generic engineering. Reproduced from [9], with permission from WILEY-VCH, 2009.

The filamentous type of M13 consists of approximately 2700 copies of the spirally arranged pVIII major coat protein on the body, with 5 to 7 copies of pIII, pVI, pIX, and pVII minor coat proteins at each end. M13 has a regular form, with a length and diameter of ~880 nm and ~6.6 nm, respectively. The genome structure and protein sequences of the M13 bacteriophage are fully understood. Therefore, M13 bacteriophages are very simple to genetically engineer to achieve the desired biochemical properties [6,7,8,9]. Figure 1 shows the basic protein structures and genetic engineering pathways of M13 bacteriophages. For example, genetically modifying the pVIII protein of M13 bacteriophage to possess the tryptophan–histidine–tryptophan (WHW) peptide sequence results in it showing specific binding affinity to nitrotoluene derivatives [4,10,11,12,13,14]. Consequently, the specific binding affinity to target materials, including chemical and biological materials, by simple genetic engineering provides great possibilities to use M13 bacteriophages as the core material of sensors.

M13 bacteriophages have a filamentous shape and different protein configurations at each end. One end has the pVI and pIII protein and the other has the pVII and pIX protein. By the well-defined, anisotropic structure and monodispersity caused by the natural character of M13, the material can be fabricated easily in hierarchical self-assembled structures using appropriate techniques [4,14]. The structure and behavior of M13 bacteriophages during fabrication are similar to those of liquid crystals. The bacteriophage can also be modified chemically and genetically to reveal a range of features, such as desired functionality and target-specific affiliation [11,12,13].

The target-specific reactivity character of the M13 bacteriophage makes it advantageous for sensor applications. The other strong point of the M13 bacteriophage is its ability to form higher-order dimensional structures in a specified order over self-assembly technology [13]. High-dimensional structures using natural building blocks, such as the M13 bacteriophage, were inspired by natural systems, such as the collagen bundle structure [4]. Although the imitation of a natural assembly structure on the micro scale using artificial building blocks, such as polymers or artificial nanomaterials, is progressing slowly, due to uncertainty and complexity, using natural building blocks such as M13 bacteriophage can be a solution. The filament structure and short dispersion of the M13 bacteriophage allow liquid crystal-like behavior in suspension under controlled conditions [15,16]. This liquid crystal behavior of the M13 bacteriophage can be managed mostly by the concentration of the M13 bacteriophage aqueous suspension. The crystal structure of self-assembled M13 bacteriophages shows nematic-, cholesteric-, and smectic-phase at low, medium, and high concentrations, respectively [16]. M13 is also attractive owing to its easy growth and handling characteristics. The use of natural substances as a building block makes a synthetic process possible at low cost under a non-toxic environment. The fabrication process associated with M13 bacteriophages using aqueous self-assembly technology allows easy processing without the need for organic solvents and additional processing. By using the pulling technique, concentration of M13 bacteriophage aqueous solution and polling speed of substrate are main factors in the process [17,18].

An additional outstanding benefit of M13 bacteriophages is the easy genetic modification for the specific binding affinity to definite target substances. Each coat protein can be modified genetically using phage display technology. Random phage libraries containing more than 1.0 × 10^11^ random peptide sequences of M13 bacteriophages are screened for specific target materials [19,20]. The binding phage on the target material is then selected and the bacteria are infected and amplified. After repeating this process to isolate the target-specific binding phage, the target-specific peptide can be recognized by DNA analysis [21,22]. Recently, the technology has developed a M13 bacteriophage that binds specifically to several inorganic resources, e.g., GaAs, GaN, Ag, Pt, Au, Pd, Ge, Ti, SiO_2_, quartz, CaCO_3_, ZnS, CdS, Co, TiO_2_, ZnO, CoPt, FePt, BaTiO_3_, CaMoO_4_, and hydroxyapatite [21,22,23,24,25]. The M13 bacteriophage can be made to have specific functionality by genetically engineering to carbon-based nanomaterials, such as C_60_, graphene, and carbon nanotubes [24,25]. Moreover, these specific binding capacities of the M13 bacteriophage can be used as templates to produce various nanostructures, such as highly monodisperse nanostructures [26,27,28]. This review introduces the current advances of M13 bacteriophage-based biosensors.

### 1.2. M13 Bacteriophage-Based Biosensor

**Figure 2:**
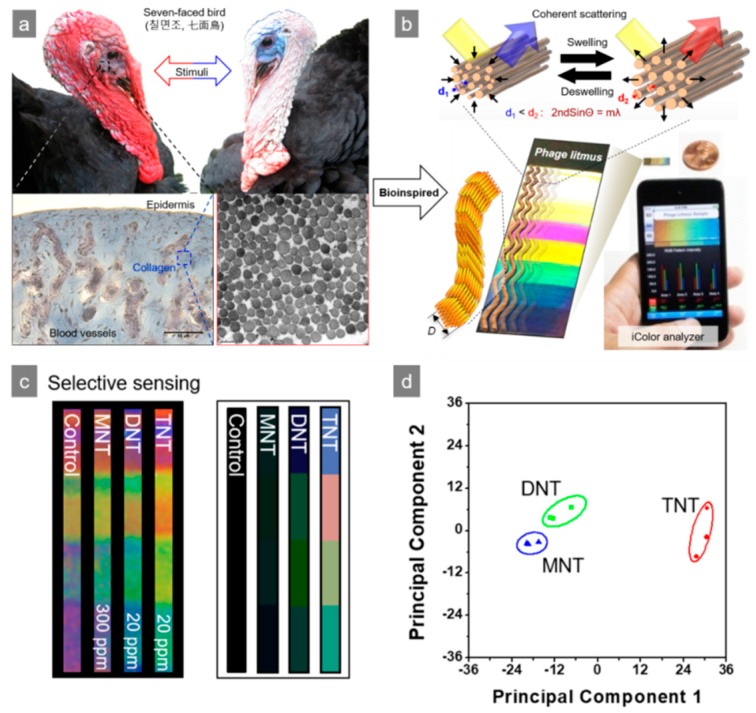
Nitrotoluene derivative detection using a M13 bacteriophage-based color sensor. (**a**) Bio-mimic structure of turkey collagen, (**b**) reversible color responding of a color sensor, (**c**) sensitivity of M13 bacteriophage-based color sensor upon exposure to nitrotoluene derivatives, and (**d**) principal component analysis (PCA) plot of the color changes. Reproduced from [4], with permission from Springer Nature, 2014.

The M13 bacteriophage has been used to improve existing sensor systems. Förster resonance energy transfer (FRET) is based on long-term bipolar interactions between excited state donors and ground state recipients. The distance of each N-terminal to the end of the peptide on the surface of a M13 bacteriophage is approximately 3.2 nm (oa) and approximately 2.3 nm (ob), respectively. Wang et al. recently reported the fabrication of FRET-based lattice probes using the M13 bacteriophage [29,30]. Owing to the regular organization of the M13 bacteriophage on the substance, a thin coating film of M13 bacteriophage can be used for surface plasmon resonance (SPR) measurements. Genetically engineered M13 bacteriophages, which possess the Arg-Gly-Asp (RGD) peptide sequence, have been used to detect the SPR signal of the cell proliferation rate and the morphology of cells [31]. The signal strength of surface-enhanced Raman spectroscopy (SERS) depends strongly on the distance between the target molecules and novel metal surface. Therefore, the controlled organization of metal nanoparticles and Raman active dye is the critical point. Cha et al. introduced an Au@Ag-core shell nanoparticle using an M13 bacteriophage as the template. In this approach, nanoparticles were functionalized by DNA-conjugated M13 bacteriophage, and showed a 75 times stronger Raman signal than DNA-functionalized nanoparticles without the M13 bacteriophage. This was mainly caused by the large number of functional moieties on pVIII protein of the M13 bacteriophage [32]. The M13 bacteriophage could also be used medically because of the strong specific reactivity to a particular reactant. Mao et al. fabricated M13–liposome–ZnPc (zinc phthalocyanine) for a more stable and upgraded cancer drug delivery system [33,34,35]. Simple genetic engineering could reveal target-specific affinity to the M13 bacteriophage. Oh et al. engineered the pVIII protein of M13 bacteriophage to have the AXXXWHWQXXDP (WHW) sequence and showed excellent binding affinity to trinitrotoluene (TNT), as shown in Figure 2a,b [36,37]. The modified M13 bacteriophage was then fabricated as a color film structure through a simple pulling technique. As shown in Figure 2c,d, the M13 bacteriophage-based color sensor could detect the gas phase of TNT down to the 300 p.p.b level and showed superior selectivity among TNT, Dinitrotoluene (DNT), and Nitrotoluene (MNT) [4].

## 2. M13 Bacteriophage-Based Protein and Microorganism Sensing

**Figure 3:**
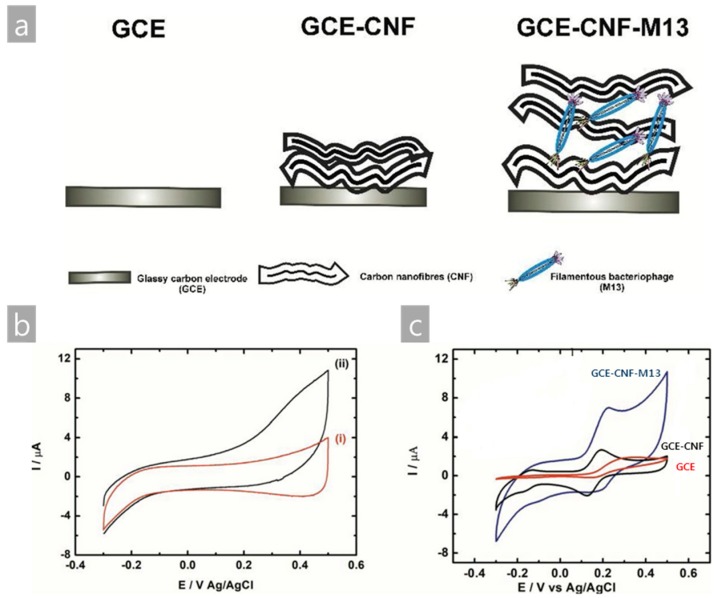
(**a**) Schematic diagram of the device fabrication of the CNF and M13 bacteriophage complex electrode, (**b**) cyclic voltammograms (3rd scan) of (i) glassy carbon electrode (GCE)–CNF and (ii) GCE–CNF–M13 bacteriophage treated in 1 mM cysteine solution in PBS, and (**c**) cyclic voltammograms (2nd scan) of the bare GCE, GCE–CNF, and GCE–CNF–M13 bacteriophage. Reproduced from [39], with permission from Elsevier, 2019.

Indeed, using a natural M13 bacteriophage without any genetic engineering provides a great opportunity because of the abundant negative charge on the surface protein. The α-helical major coat protein (pVIII) has glutamate amino acid on the end of its sequence. Therefore, the dipole moment of the whole pVIII protein is directed from the outside (*N*-terminus) to the core (*C*-terminus). The charge distribution of the *C*-terminus (positive) and *N*-terminus (negative) induces a strong dipole to M13 bacteriophages possessing a natural negative charge [38]. This property allows the M13 bacteriophage to mix easily with positively charged materials, such as carbon nanofiber (CNF). Recently, Niedziòłka-Jönsson et al. reported a M13 bacteriophage and CNF complex structure for cysteine detection. The M13 bacteriophage and CNF were mixed in an aqueous solution, and it was assumed that the electrostatic interaction and π-π interaction between the M13 bacteriophage and CNF promoted an even distribution of CNF without aggregation, which occurred constantly when CNF was used alone in solution [39,40,41]. As a result, the addition of M13 bacteriophage to CNF increased the capacitive current with the growth of the faradaic current. An overall improvement of the electrochemical properties of the glassy carbon electrode (GCE)–CNF–M13 bacteriophage complex electrode applied to the electrocatalytic oxidation of the cysteine was observed and enhanced results were obtained, as shown in Figure 3 [39]. The fabrication in this study simply dropped CNF solution on the substrate (indium tin oxide (ITO) glass) then M13 bacteriophage dropped on the dried CNF. Although the process is not yet optimized, the study shows promising results. Using wild type M13 bacteriophage for this experiment was another strong point due to the simple preparation.

**Figure 4:**
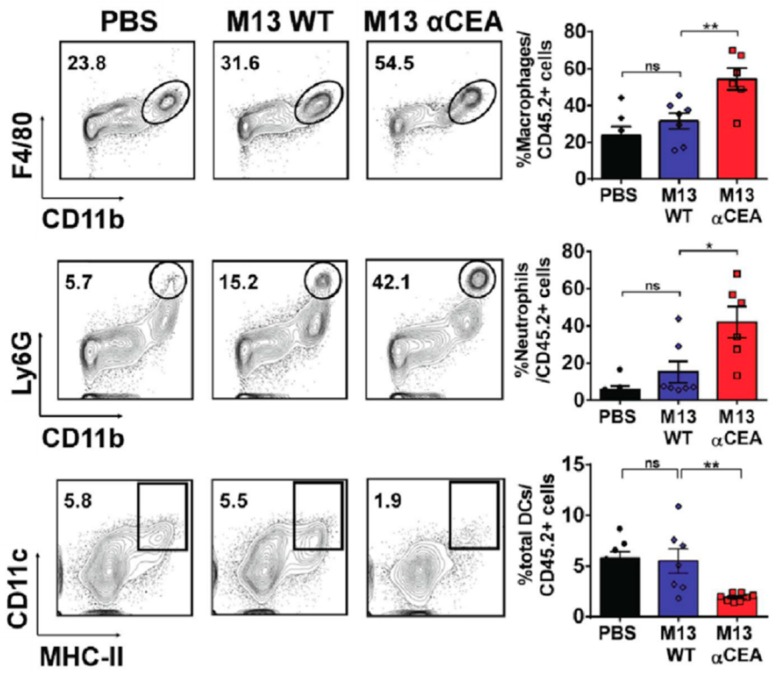
Treating the tumor microenvironment using PBS, wild type M13 bacteriophage, and αCEA-M13 bacteriophages for 24 h. Contour plots shows tumor infiltrating macrophages (F4/80^+^CD11b^+^), (**upper left**), neutrophils (CD11b^+^Ly6G^high^), (**middle left**), and dendritic cells (CD11c^+^MHC-II^+^), (**lower left**). The percentage of each plot (**right-hand panel**). Reproduced from [44], with permission from Springer Nature, 2017.

**Figure 5:**
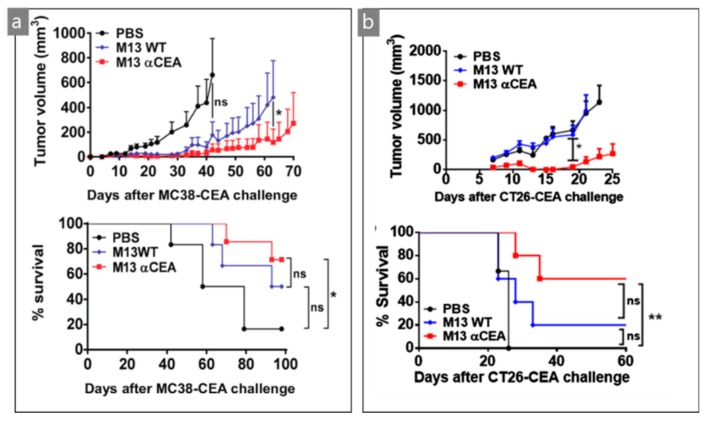
Growth of different CEA tumors ((**a**) MC38-CEA, (**b**) CT26-CEA) and survival of mice treated with M13 bacteriophage with or without αCEA. Reproduced from [44], with permission from Springer Nature, 2017.

As mentioned above, simple genetic engineering provides a specific interaction to a designated target material. Lladser et al. genetically engineered colorectal cancer (CRC)-specific M13 bacteriophages. The M13 bacteriophage was modified to have carcinoembryonic antigen (CEA)-specific moiety. CEA exists abundantly in colorectal cancer and is known to support the malignant features of colorectal cancer cells such as cell adhesion, cell-to-cell interaction, and signal transduction [42,43]. The CEA level clearly has metastatic potential, cancer progression, differentiation, and apoptosis of CRC cells [44,45,46]. In this study, CEA-specific (αCEA) M13 bacteriophages were applied to tumor cells for the tumor infiltration of neutrophils, macrophages, and the maturing of dendritic cells in tumor-draining lymph nodes [44]. For M13 bacteriophage genetic engineering, they transformed *E. coli* with a pSEX81 surface expression phagemid vector, which possesses CEA-specific single-chain fragment variable (scFv). The scFv was inserted at the NcoI and BamHI restriction sites upstream of the pIII protein sequence. *E. coli* were infected with a multiplicity of infection by a hyperphage that contains all genes of structural proteins of M13 bacteriophage except for the pIII protein [47]. The αCEA-M13 bacteriophage exhibited strong binding affinity to both CEA and CEA-expressing tumor cells (CT26) in vitro. The applied αCEA-M13 bacteriophage effectively suppressed a mouse cancer model compared to the phosphate-buffered saline (PBS)-control and wild type M13 bacteriophage experiment by successful intratumoral and systemic administration. Here, they confirmed that the tumor protection provided by αCEA-M13 bacteriophage occurred via CD8^+^ T cells because the reduction of circulating CD8^+^ T cells completely eliminated the antitumor protection. Figure 4 shows that the macrophage (F4/80^+^) increased from approximately 24% to 54%; the neutrophils increased from approximately 6% to approximately 42%, and the tumor-infiltrating dendritic cells decreased from approximately 6% to approximately 2%. In other words, the application of αCEA-M13 bacteriophages to tumor cells resulted in an increase in the tumor infiltration of innate immune cells and maturing of dendritic cells at lymph nodes. Figure 5 shows decreased tumor volume and increased survival rate of mice by treating with αCEA-M13 bacteriophage. Their studies can be regarded as a successful suggestion of potential immunotherapy against CRC [44]. Antigen–antibody reaction is one of the most simple and widely used treatment methods in the medical field. However, it is still the most useful clinical method. Using highly concentrated functional moiety on the M13 bacteriophage, these types of treatments can be more effective as immunotherapy against CRC.

**Figure 6:**
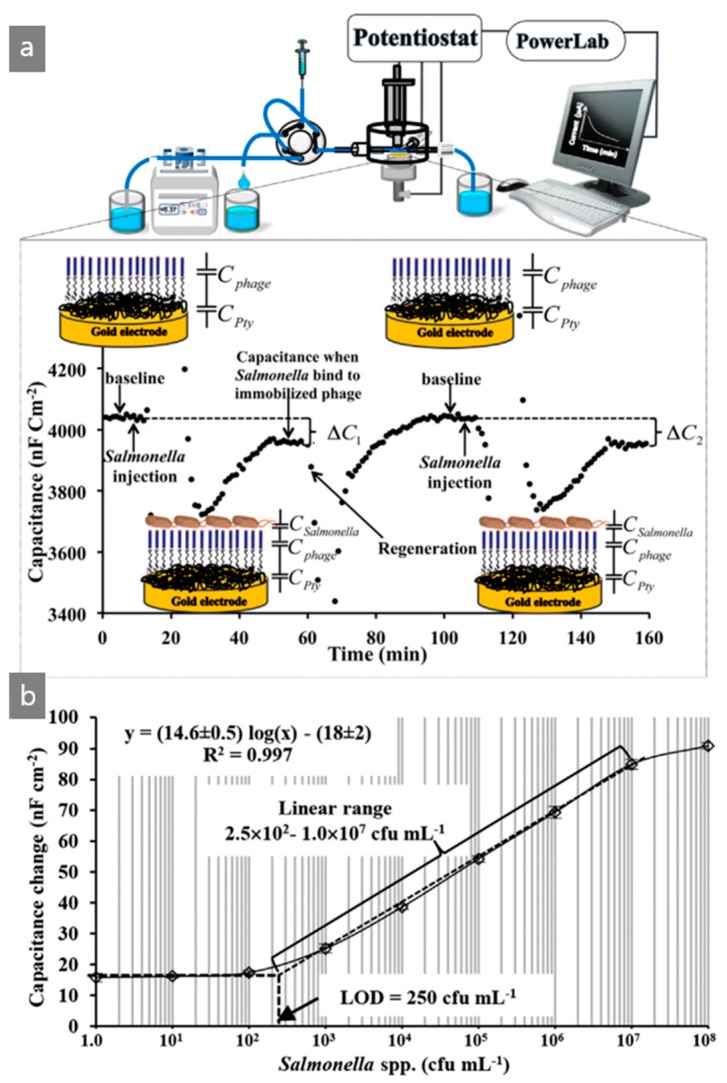
(**a**) Schematic procedure of the capacitive flow injection system for sensing *Salmonella* spp. using a M13 bacteriophage-based electrode sensor and (**b**) linear response of the sensor by number of *Salmonella* spp. Reproduced from [48], with permission from Elsevier, 2018.

*Salmonella* detection using a M13 bacteriophage is one of the largest fields of M13 bacteriophage research and could feasibly replace conventional methods, such as the polymerase chain reaction (PCR) and immunology-based assays [48,49,50]. Among the many types of pathogenic bacteria, *Salmonella* detection has received more attention because of its prevalence [51]. Recently, Thavarungkul et al. described a capacitive flow injection system for *Salmonella* spp. detection using a *Salmonella*-specific M13 bacteriophage in a working electrode [48]. They genetically modified the wild pVIII protein to have the NRPDSAQFWLHHGG sequence based on the phage display technique. They simply placed the *Salmonella*-specific M13 bacteriophage on a polytyramine (Pty)-coated gold electrode, and the amount of injected target *Salmonella* was determined by the capacitance of *Salmonella* over the total capacitance. Figure 6a shows the overall sensing procedure of this sensor device. The M13 bacteriophage-based electrode sensor was located in the running flow buffer. When the target *Salmonella* spp. was injected, the capacitance rolled up and down, and the new capacitance could be obtained. This capacitance was lower than that without a reaction to *Salmonella*. The difference in capacitance is denoted as ΔC and the total capacitance could be calculated as follows:

(1) $$\frac{1}{C_{total}}=\frac{1}{C_{Pty}}+\frac{1}{C_{phage}}+\frac{1}{C_{Slamonella}}$$

The M13 bacteriophage-based electrode sensor was then washed with a recovery solution (5.0 mM of NaOH solution) to break the binding between the M13 bacteriophage and *Salmonella*. The M13 bacteriophage-based *Salmonella* sensor showed great selectivity to the *Salmonella* samples and exhibited an excellent recovery ratio, almost 100%. As a result, in Figure 5b, the sensor showed good sensing capability to detect *Salmonella* in chicken samples with linear range from 1.0 × 10^3^ to 1.0 × 10^7^ cfu/mL [48]. *Salmonella* detection is one of the main M13 bacteriophage biosensor research areas. Meanwhile, this research was performed using magnetoelastic, SPR, and microcantilever technique. The capacitive flow system was introduced very recently, and showed great results.

**Figure 7:**
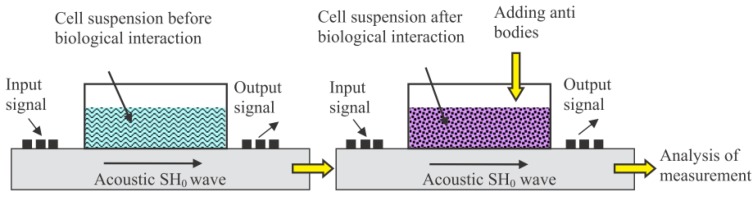
Schematic diagram of the electroacoustic measurements using an X–Y lithium niobate piezoelectric plate and target cell-specific M13 bacteriophage solution. Reproduced from [52], with permission from Elsevier, 2018.

**Figure 8:**
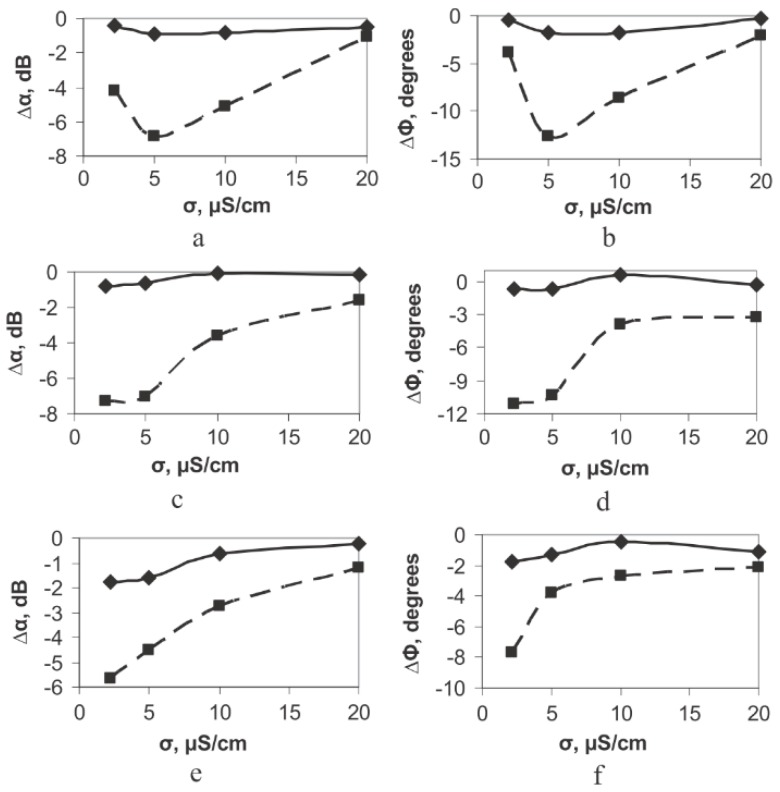
The change in the insertion loss (∆α, left) and phase of the output signal (∆Φ) of the conductivity of the buffer solution. (**a**,**b**) 10^4^ cells/mL, (**c**,**d**) 10^6^ cells/mL, and (**e**,**f**) 10^8^ cells/mL. The solid and dotted lines correspond to the electrically open and shored channels, respectively. Reproduced from [52], with permission from Elsevier, 2018.

The target-specific binding affinity of the M13 bacteriophage could alter the macro-structure or morphology as well as the charge density of the M13 bacteriophage suspension. Therefore, the acoustic signal that passes through the M13 bacteriophage suspension can indicate the target-specific reaction both quantitatively and qualitatively [52,53,54]. Formin et al. reported an M13 bacteriophage-based biosensor for the detection of microbial cells with antibodies on the M13 bacteriophage [52,53]. They reacted initially with the specific antibody on the M13 bacteriophage to have a specific interaction with the target cell line (*Azospirllum basilense* strain Sp245). The cell suspension was located directly on the thin piezoelectric plate, which propagates the piezoactive acoustic wave by the input signal. Figure 7 presents a schematic diagram. After a specific interaction of M13 bacteriophages with the target microbial, they could measure significant insertion loss and phase change by a reaction. The parameters were also changed sensitively by different microbial numbers. Figure 8 shows increase conductivity of the solution caused a decrease in change of the phase and insertion loss of the output signal by increasing the number of cells. By increasing cell numbers, the interaction between M13 bacteriophage and cells occurs. Resulting clear signal changes were observed from 10^4^ to 10^7^ cells/mL [52]. Thus far, this is one of a few results using electroacoustic techniques to detect microbial target-specific interaction. Since this technique can be performed directly in suspension, it can be a useful supplement method for existing methods.

## 3. M13 Bacteriophage-Based Chemical Sensing

The M13 bacteriophage has a systemically regular shape because it is a natural substance. The highly certain shape and specific reactivity to the target material provided to the M13 bacteriophage have great potential to become a building template [6]. Haberer et al. discussed the gold nanopeapod structure covered with polypyrrole (PPy) located directly between platinum electrodes. In this particular experiment, an M13 bacteriophage was genetically engineered to have the VSGSSPDS protein sequence on its pVIII major coat protein. A gold-specific M13 bacteriophage was covered and formed a bridge between two pre-fabricated platinum electrodes, and then reacted with a gold solution. A commercial gold electroless deposition reagent was used to increase the size of the gold nanoparticle. Finally, electrodeposition of PPy was conducted to complete the gold PPy nanopeapod structure between the electrodes [55,56]. The product was applied as an NH_3_ gas sensor due to nucleophilic NH_3_ attack of the polymer backbone, decreasing the conjugation length, and irreversibly increasing the resistance. In other words, NH_3_ molecules in this reaction act as an electron donor. Consequently, decreasing the electron hole concentration of the polymer results in a reversible de-doping process [57,58,59]. In this experiment, the irreversible reaction has competitive advantage over the reversible reaction, and partial recovery after gas sensing was achieved. This study showed an excellent detection limit of 0.005 ppm by NH_3_ exposure to a M13 bacteriophage template gas sensor [55]. This technique implies resistance of one-dimensional nanowires which were fabricated using M13 bacteriophage as gas sensor. Due to the extremely thin layer of PPy and the catalytic effect of Au NPs, this technique showed great sensitivity. This structure highly depends on random connection between electrodes. Better performance can be expected from this structure by optimized structure such as nano pattern between two electrodes.

**Figure 9:**
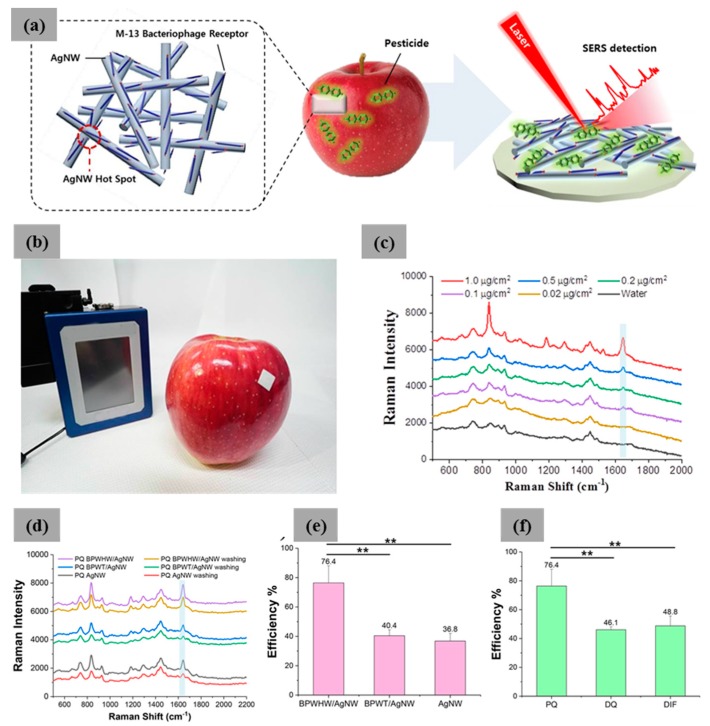
(**a**) Illustration of surface-enhanced Raman spectroscopy (SERS) by a functionalized M13 bacteriophage, (**b**) practical application of an M13 bacteriophage-based SERS sensor on an apple surface, (**c**) Raman spectra of paraquat (PQ) at different PQ concentrations on an apple surface, (**d**) Raman spectra of PQ using tryptophan–histidine–tryptophan (WHW), wild type of bacteriophage and without M13 bacteriophage before and after the washing step, (**e**) efficiency comparison of WHW, wild type and nanowire only, and (**f**) efficiency comparison of different pesticide (PQ, diquat (DQ), and difenzoquat (DIF)) using WHW M13 bacteriophage. Reproduced from [60], with permission from American Chemical Society, 2018.

SERS technology based on the nanowire structure can be improved significantly by controlling the density of “hot spots”, where Raman signal amplification occurs [60,61] For more effective signal amplification, the sensor surface needs to be highly functionalized, particularly around hot spots [62,63]. Jung et al. recently reported an M13 bacteriophage-covered silver nanowire SERS sensor to detect a certain pesticide, paraquat (PQ), which is a widely used herbicide. The M13 bacteriophage was functionalized genetically to have the WHW peptide sequence for the PQ specific binding affinity on its pVIII major coat protein. When the sensor was compared with the wild type M13 bacteriophage sensor and a sensor without M13 bacteriophage, the genetically functionalized sensor showed superior selectivity to the PQ sample [60]. They also tested the selectivity upon different pesticides of a functionalized M13 bacteriophage-SERS sensor using bipyridylium, which is a common herbicide. The WHW-type M13 bacteriophage-functionalized SERS sensor showed a better Raman signal when PQ was detected than bipyridylium at the same concentration. In Figure 9, using a hand-held Raman spectrometer, they could detect PQ concentrations as low as 0.1 μg/cm^3^, which is far below the guidelines of most countries. Raman intensity changes before and after the washing step showed best results for WHW type M13 bacteriophage sensors. It also showed better efficiency when compared with wild type and nanowire without M13 bacteriophage. By measuring different pesticides (diquat (DQ) and difenzoquat (DIF)), PQ was the most suitable pesticide. [60,64]. This practical research showed detection results using a hand-held Raman spectrometer and agricultural product without pretreatment.

## 4. M13 Bacteriophage-Based Color Sensor

**Figure 10:**
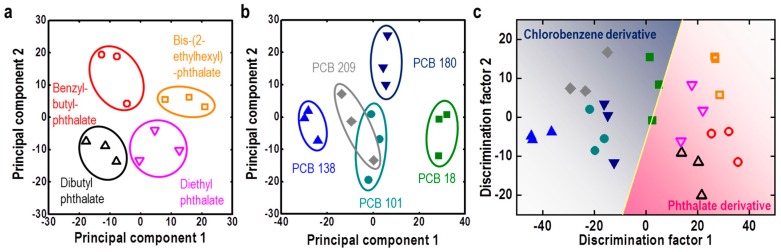
(**a**) PCA analysis of phthalate derivatives, (**b**) PCB derivatives, and (**c**) LDA analysis of phthalate and PCB derivatives using a M13 bacteriophage-based color sensor. Reproduced from [65], with permission from Elsevier, 2017.

The color sensor is one of the fields of M13 bacteriophage research, which has great potential in terms of sensitivity, selectivity, and portability [4,65,66]. Endocrine-disrupting chemicals were detected using an M13 bacteriophage-based color sensor. Genetically engineered M13 bacteriophages can be manufactured with a color sensor that has a structural color using a simple pulling technique, and applied to endocrine disrupting substance detection [65]. Although existing research focused on one specific substance, such as TNT [NC 13], this research focused on classifying various types of endocrine disruptors with similar structures. In this study, polychlorinated biphenyl (PCB) and phthalate compounds, traditional flame retardants, and plasticizers, were selected. These substances, which have been selected as regulated substances, are still included in pre-regulated polymer products, which act as environmental pollutants for use and disposal [65,67]. The M13 bacteriophage-based structural color sensor was modified with phage display technology. The protein sequence genetically engineered on the pVIII major coat protein is specific for covalent binding and is effective in the detection of aromatic compounds. In this study, four phthalate compounds and five PCB compounds were distinguished with high sensitivity, and the entire phthalate and PCB compound groups were distinguished with high accuracy (Figure 10) [65]. The detection limit of this color sensor was approximately 100 ppm, and the first two principal components of PCA analysis accounted for 54.89% of phthalate derivatives and 67.38% of PCB derivatives. The linear discriminant analysis (LDA) error between phthalate and PCB derivatives was 3.7% [65,68].

**Figure 11:**
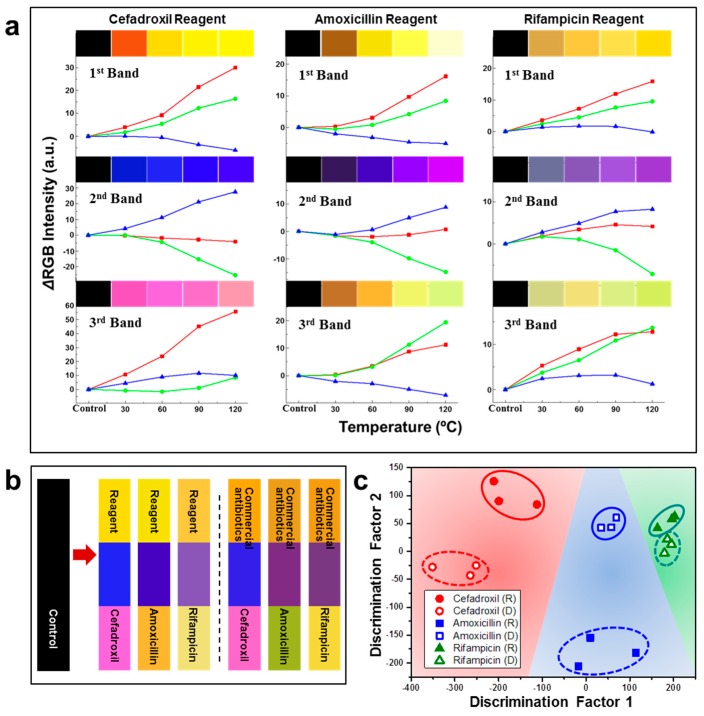
Measurement of various antibiotics using M13 bacteriophage-based color sensors. (**a**) Sensitivity measurement at different temperatures using fixed amount of samples, (**b**) color change translation of samples, and (**c**) PCA analysis of antibiotic sensing. Reproduced from [69], with permission from Wiley-VCH, 2016.

Drug abuse causes serious health and environment problems [69,70,71]. Color sensor systems using M13 bacteriophages have shown promise for the detection of toxic substances [4,65]. Owing to the specific selectivity and sensitivity to target materials, M13 bacteriophage-based color sensors have the potential to detect various drugs [69]. Oh et al. discussed the structural color sensor to distinguish a range of antibiotics. The M13 bacteriophage-based structural color sensor was manufactured by inducing self-assembly using a simple pulling technique. Briefly, substrate (Au coated Si wafer) was deepen into M13 bacteriophage aqueous solution and pulled out slowly. By controlling the concentration of solution and pulling speed, a structural color band could be achieved through evaporation of M13 bacteriophage solution [4,65,69]. When the color sensor was exposed to an organic solvent, the bacteriophage bundle nanostructure constituting the microstructure of the sensor expanded, resulting in a structural color change. A genetically engineered M13 bacteriophage surface protein structure called WHWQ in this study could clearly distinguish three types of commercial antibiotics and three types of core ingredients of these antibiotics, which were confirmed by color and PCA analysis. Cefadroxil, amoxicillin, and rifampicin are some of the most popular antibiotics that a large number of pharmaceutical companies produce, and an absolute majority of patients consume. In Figure 11, PCA analysis produced clear discrimination between the three types of antibiotics as well as commercial and reagent grade. The first two discriminant factors accounted for 86.89% in this study [69].

**Figure 12:**
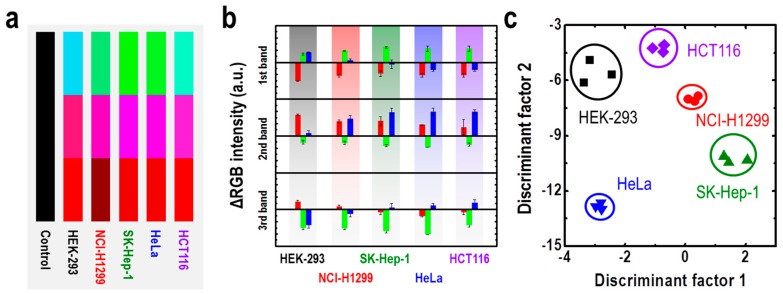
Cancer cell type identification using M13 bacteriophage-based color sensors. (**a**) Red, Green, and blue (RGB) color patterns of each cancer cell, (**b**) RGB intensity change, and (**c**) PCA analysis of samples. Reproduced from [72], with permission from The Royal Society of Chemistry, 2017.

An M13 bacteriophage-based color sensor also could be applied to microbial detection. Oh et al. reported a color sensor capable of detecting cancer cell types based on the M13 bacteriophage [72]. The M13 bacteriophage-based color sensor works in the form of a photonic nose, which functions similarly to other researched electronic noses, and shows structural color-based color changes by contact with the specific target materials. The target material in this study was a specific volatile compound that is unusually abundant in various cancer cells. The respiratory by-products of cancer cells, which are distinguished uniquely from normal cells, include hydrazine, xylene, ethylbenzene, ethanol, and toluene [72,73]. Therefore, they measured a range of organic reagents, populations of *E. coli*, and CO_2_ concentration using the M13 bacteriophage-based color sensor. Here, the M13 bacteriophage-based color sensor showed significant sensitivity and selectivity for organic solvents, cell population, and CO_2_ concentration. In this study, selectivity experiment has been done for hydrazine, xylene, ethylbenzene, ethanol, and toluene, volatile organic chemicals. These chemicals can be found in abnormally high concentration of in the lung cancer patient’s respiration [72,73,74,75]. These organic compounds can act like an analytic marker for different cancer cells when released through respiration of cancer cells. Therefore, selective detection of these organic solvents was important to the selective detection of various cancer cells. Finally, each cancer cell’s respiration by-product was analyzed using adjacent color sensors while culturing various types of cancer cells. As a result, they showed excellent discrimination through PCA and color analysis, with the first two discriminant factors accounting for 99.8% (Figure 12) [72].

## 5. Expansion of Analytic Method from Direct Sensing Using M13 Bacteriophage

**Figure 13:**
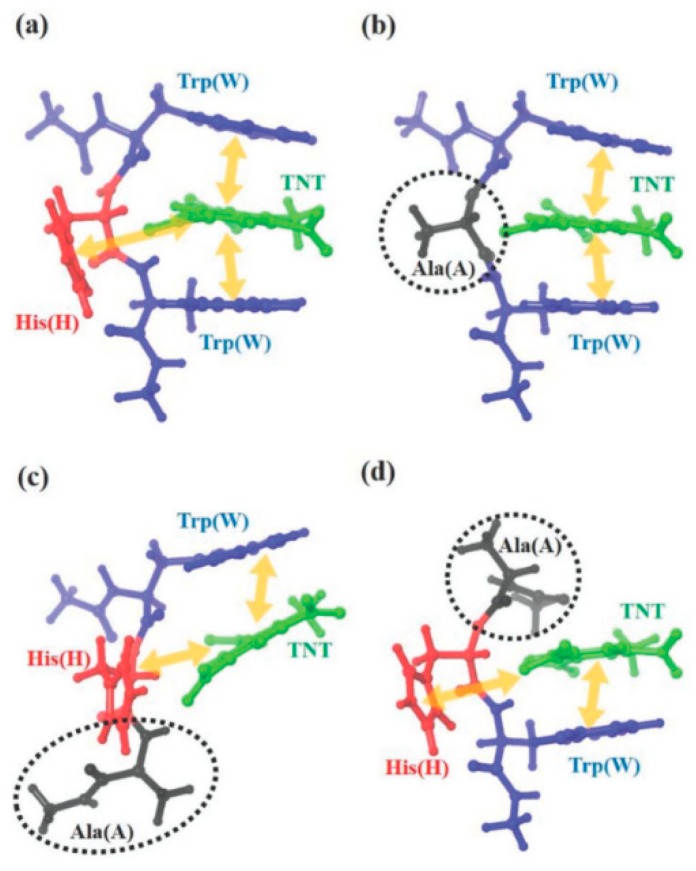
The stable constructions of TNT-specific peptides and their reactivity (red: histidine, blue: tryptophan, black: alanine, green: TNT, yellow, and arrow: π-π interaction). (**a**) WHW–TNT, (**b**) WAW–TNT, (**c**) WHA–TNT, and (**d**) AHW–TNT. Reproduced from [76], with permission from The Royal Society of Chemistry, 2019.

A range of variations of WHW type M13 bacteriophages have common specific affinity to TNT molecules [4]. A π-π interaction was assumed among TNT molecules, histidine, and tryptophan based on experiment [4,6]. On the other hand, there is a lack of consideration as to whether WHW is really the best structure for TNT molecules or how this specific interaction occurs systemically and theoretically. Recently, Lee et al. reported the results of computational calculations of this interaction among TNT molecules, histidine, and tryptophan, using quantum mechanics (QM) calculations [76]. The theoretical candidates for comparison were WHW-, WAW-, WHA-, and AHW-simulated peptide sequences (A: Alanine). The specific binding affinity between the WHW and TNT molecules was confirmed experimentally using an SPR sensor system due to the significantly lower detection limit (500 fM) than any other sensor system based on M13 bacteriophages [4,77,78,79,80]. The dissociation constants for TNT molecules using the WHW-, WAW-, WHA-, and AHW-type M13 bacteriophages were 7.0 × 10^−12^, 2.7 × 10^−11^, 1.1 × 10^−10^, and 3.3 × 10^−8^, respectively. By QM calculations using Jaguar v8.4 software with the M06-2X/6-31G ** level of density functional theory, the binding energy of WHW-TNT was calculated to be 22.7 kcal mol^−1^. Figure 13 shows the active binding mechanism for WHW and alanine substituted peptide sequences. For WAW, WHA, and AHW, the binding energy was 19.9, 16.0, and 18.8 kcal mol^−1^, respectively. Finally, they confirmed the strong linear correlation between the experimental Gibbs free energy and calculated binding energy [76].

## 6. Conclusions

M13 bacteriophages have attracted considerable attention because of their simplicity of structure and functionality. The additional merit of M13 bacteriophages is the customized functionality, which can be provided easily through simple genetic engineering. In Table 1, large numbers of research groups have examined various fields using M13 bacteriophages. In particular, a biosensor using M13 bacteriophages has attracted serious interest because of the portability and intuitiveness. M13 bacteriophage has been utilized as an electrode support material for cysteine and *Salmonella* spp. detection [39,44,48]. Functionalized M13 bacteriophages have the potential to detect various cancer cells effectively [44,72]. Acoustic and SERS signals could be amplified by adding M13 bacteriophages to existing sensor systems [52,60]. In addition, M13 bacteriophage-based color sensors have been evaluated as a key tool to apply M13 bacteriophages to sensor systems [65,69,72]. Application of the M13 bacteriophage is still in its infancy. There remain many more protein sequences to be found, and low dimensional fabrication control should be studied. Mass production and reproducibility of nanostructures also need to be studied and overcome. Active theoretical studies on the function of genetically engineered M13 bacteriophages for specific target materials will open more systematic approaches as well as novel applications of M13 bacteriophages [12,76].

## Figures and Tables

**Table 1:** Biosensor applications of M13 bacteriophages.

M13 Bacteriophage Immobilizations	Detection Technique	Analytes	Real Sample	Ref.
M13/Au coated Si wafer	Color analysis	Nitrotoluene	TNT, DNT, MNT	[4]
M13/donor and acceptor dye	FRET	Intracellular pH	RAW264.7 macrophage	[30]
M13/Au coated glass	SPR	Cell proliferation signal	NIH3T3 mouse fibroblast	[31]
M13/Au@Ag NPs/Raman dye/DNA	SERS	Antibody concentration	Sterptavidin/anti-goat IgG	[32]
M13/zinc phthalocyanine/methyl viologen	Fluorescence image	Breast cancer cells	SKBR-3 cell line	[35]
M13/CNF/GCE	Electrochemical	Cysteine	L-Cysteine solution in PBS	[39]
M13/scFv	Optical/surgical	CEA tumor cells	MC38- and CT26-CEA	[44]
M13/Pty/Au electrode	Capacitive	Salmonella	Chicken	[48]
M13/antibody	Electroacoustic	Cell number	Sp245 cell line	[52]
M13/Ag nanowire	SERS	Pesticides	PQ, DQ, and DIF	[60]
M13/Au coated Si wafer	Color analysis	Endocrine disrupting chemicals	Phthalate and PCB derivatives	[65]
M13/Au coated Si wafer	Color analysis	Antibiotics	Commercial and reagent antibiotics	[69]
M13/Au coated Si wafer	Color analysis	Cancer cells	HEK-293, NCI-H1299, SK-Hep-1, HeLa, HCT116 cancer cell lines	[72]
